# Isolation and Characterization of Three Mammalian Orthoreoviruses from European Bats

**DOI:** 10.1371/journal.pone.0043106

**Published:** 2012-08-14

**Authors:** Claudia Kohl, René Lesnik, Annika Brinkmann, Arnt Ebinger, Aleksandar Radonić, Andreas Nitsche, Kristin Mühldorfer, Gudrun Wibbelt, Andreas Kurth

**Affiliations:** 1 Robert Koch Institute, Centre for Biological Security 1, Berlin, Germany; 2 Leibniz Institute for Zoo and Wildlife Research, Berlin, Germany; University of Ottawa, Canada

## Abstract

In recent years novel human respiratory disease agents have been described in South East Asia and Australia. The causative pathogens were classified as pteropine orthoreoviruses with strong phylogenetic relationship to orthoreoviruses of flying foxes inhabiting these regions. Subsequently, a zoonotic bat-to-human transmission has been assumed. We report the isolation of three novel mammalian orthoreoviruses (MRVs) from European bats, comprising bat-borne orthoreovirus outside of South East Asia and Australia and moreover detected in insectivorous bats (*Microchiroptera*). MRVs are well known to infect a broad range of mammals including man. Although they are associated with rather mild and clinically unapparent infections in their hosts, there is growing evidence of their ability to also induce more severe illness in dogs and man. In this study, eight out of 120 vespertilionid bats proved to be infected with one out of three novel MRV isolates, with a distinct organ tropism for the intestine. One isolate was analyzed by 454 genome sequencing. The obtained strain T3/Bat/Germany/342/08 had closest phylogenetic relationship to MRV strain T3D/04, isolated from a dog. These novel reoviruses provide a rare chance of gaining insight into possible transmission events and of tracing the evolution of bat viruses.

## Introduction

Since most of the recent disease outbreaks have been associated with zoonotic transmission events of newly emerging viruses, it has become evident that surveillance and evaluation of viruses prevalent in wildlife are of particular importance [1]–[2]. Bats are increasingly known to be reservoir hosts of various viruses [3]. Since Gard et al. reported the first isolation of a novel orthoreovirus from a fruit bat in Australia (Nelson Bay) in 1973, bats are likewise known to be possible carriers of reoviruses [4]. Later, while searching for the natural reservoir host of Nipah virus, another bat-borne orthoreovirus called Pulau virus was isolated from a fruit bat’s urine on Tioman Island, Malaysia [5]. In the past five years bat-borne orthoreoviruses again received attention after a novel respiratory disease agent termed Melaka virus was isolated from human patients in Malaysia in 2007 [6]. A possible zoonotic bat-borne transmission was assumed for Melaka virus, a close relative of Pulau virus, and further evidence was obtained by phylogenetic calculations. Since then five additional orthoreoviruses (Xi-River, Kampar, Sikamat, HK23629/07 and Broome virus) have been isolated from fruit bats [7]–[8] or from humans with assumed contact to bats [9]–[11]. Remarkably, all these viruses, except Broome virus, appear in one distinct phylogenetic cluster [8]. This phylogenetic species (formerly known as Nelson Bay species) was recently proposed to be renamed as *Pteropine orthoreoviruses* within the genus *Orthoreovirus* [11], while Broome virus seems to represent the first member of a novel, slightly remote cluster [8].

In general, the family *Reoviridae* can be divided genetically into two subfamilies: the *Sedoreovirinae* with six and the *Spinareovirinae* with nine comprised genera. *Orthoreovirus* is one genus within the *Spinareovirinae* and consists of five virus species: *Pteropine orthoreovirus*, *Avian orthoreovirus*, *Reptilian orthoreovirus*, *Baboon orthoreovirus* and the type species *Mammalian orthoreovirus* (MRV) which includes the orthoreoviruses of man and the majority of mammals. Orthoreoviruses are non-enveloped viruses with a segmented double-stranded RNA genome [12]. Each orthoreovirus particle contains ten genome segments, named after their migration in gel electrophoresis separation dependent on size: L1, L2, L3 (large), M1, M2, M3 (medium) and S1, S2, S3, S4 (small). The virions have an average size of 70–80 nm with a typical icosahedral, double-layered protein capsid structure.

The species MRV includes four prototype strains: type 1 Lang (T1L), type 2 Jones (T2J), type 3 Dearing (T3D) and type 4 Ndelle (T4N) [12]–[13]. These prototype strains show no immunogenic cross-reactivity with serologic differentiation. Most MRV strains can be assigned to one of the serogroups T1L, T2J and T3D. An alternative typing of novel strains is common via genetic comparison of the S1 segment which most probably encodes proteins that are responsible for serotype and tissue tropism [14].

In this study we report the isolation of three novel MRV strains from organ tissue of European bats. These bat-borne MRVs occur beyond the species *Pteropine orthoreovirus*, in insectivorous bats and outside of South East Asia and Australia. By phylogenetic analysis of the S1 segment a close, monophyletic relationship was observed to strain T3D/04 isolated from a dog suffering from hemorrhagic diarrhea and a simultaneous concurrent canine parvovirus type 2 infection. Although MRVs were assumed to cause rather mild respiratory or gastrointestinal diseases, recent findings indicate the occurrence of higher virulent MRV strains in man and other mammals [15]–[17]. The recent isolation of a novel bat virus, bat Adenovirus 2 [18], which closest phylogenetic relative is able to cause severe disease like hemorrhagic enteritis in canids, prompted us to further investigate these novel orthoreovirus isolates. This included the generation, annotation and phylogenetic analysis of the complete genome of one isolate.

## Methods

### Study

Since all German bats are covered by species protection through the European Commission (http://ec.europa.eu/environment/nature/legislation/habitatsdirective) and through the Agreement on the Conservation of Populations of European Bats (www.eurobats.org), investigative research requires special permission by local government bodies [19]. As part of a study on diseases in native bats [19], 120 bats of fourteen vespertilionid bat species were examined. (*Myotis mystacinus* [n = 21], *Pipistrellus pipistrellus* [n = 21], *P. nathusii* [n = 16], *Vespertilio murinus* [n = 11], *M. daubentonii* [n = 10], *Eptesicus nilssoni* [n = 10], *Plecotus auritus* [n = 8], *Nyctalus noctula* [n = 7], *P. kuhli* [n = 7], *E. serotinus* [n = 5], *M. bechsteinii* [n = 1], *M. brandti* [n = 1], *M. myotis* [n = 1], *M. nattereri* [n = 1]). Animals were found dead, injured or moribund near roosting sites or human habitations [19] in urban and suburban areas of different regions in Germany (Bavaria [n = 85], Lower Saxony [n = 18] and Berlin greater metropolitan area [n = 17]). All bat carcasses were kindly provided by bat researchers and bat rehabilitation centres from the different geographic regions. Permits to investigate carcasses of deceased bats were granted by the respective local governmental authorities (district government of Upper Bavaria, Munich; district government of Bavarian Swabia, Augsburg; Lower Saxony water management, coastal defence and nature conservation, Hannover; senate department for urban development and the environment, Berlin). If bats died in care or had to be euthanized for medical reasons, the carcasses were immediately stored at −20°C and transferred to the Leibniz Institute for Zoo and Wildlife Research, Berlin, Germany, for histopathological and bacteriological examination [19]. Subsequently, aliquots of the individual organs were sent to the Robert Koch Institute for virological examination.

### Pathological Investigation

A full necropsy was performed on each bat, followed by histopathological examination. Small slices of multiple organ tissues were fixed in 4% buffered formalin, processed using standard methods and embedded in liquid paraffin. Sections were cut at 2–5 µm and stained with hematoxylin-eosin.

### Virological Investigation

Vero E6 and Vero B4 cell lines were used for cell-culture screening. Cells were seeded into 24-well cell-culture plates and incubated overnight at cell-culture conditions (5% CO_2_, 37°C) to 80–90% confluence of cell monolayer. For infection, homogenates of all particular organs available per bat were pooled and incubated on either Vero E6 or Vero B4 cells for one hour at cell-culture conditions. Subsequently, the cells were sub-cultured three times, once a week and observed daily for the occurrence of cythopathogenic effects (CPE). To exclude and avoid bacterial contamination, the supernatant was passed through a 0.22 µm filter and cells were treated with a 10-fold dose of antibiotics (penicillin, streptomycin). By CPE occurrence in the third sub-cultivation, the supernatant was passed to an 80–90% confluent 175 cm^2^ flask of fresh Vero cells and incubated at cultivation conditions for one week. To harvest virus particles, cells were homogenized by three freeze-thaw cycles and the resulting suspension was purified from cell debris by low-speed centrifugation. Aliquots were stored at −80°C. One aliquot was titrated on Vero E6 cells to estimate a titre.

For live cell imaging, Vero E6 cells were seeded in a cell culture dish. Cells were infected with a MOI (multiplicity of infection) of 0.1 with strain T3/Bat/Germany/342/08. An additional uninfected dish was prepared similarly and used as a negative control. To improve visualization Opti-MEM media without phenol red (Invitrogen Life Technologies, Germany) was used during live cell imaging. Both cell culture dishes were incubated at cultivation conditions on a NIKON Eclipse TE2000-E live cell microscope and images of both cells were captured every 15 minutes for a period of 72 hours.

### Molecular Biological Investigation

DNA/RNA extraction was performed using the PureLink™ Viral RNA/DNA Mini Kit (Invitrogen, Darmstadt, Germany) on all homogenized and pooled bat organs available. For cDNA synthesis the TaqMan® Reverse Transcription Reagents kit (Applied Biosystems, Foster City, CA, USA) was utilized with an additional denaturing step of 95°C for 5 min. Bat species were determined by amplification and sequencing of mitochondrial DNA as described by Sonntag et al. [20]. For detection of reoviral RNA in internal organs a real-time PCR assay was developed, using the following primer and probe sequences: BatReoF (5′-CACCATgTCAAgCTgCTCCC-3′), BatReoR (5′-ACCgCCATgTATgTCCTCCAg-3′) and BatReoProbe (5′-6FAM-CCCAgTCgCggTCATTACCACTCCg-BBQ-3′). The PCR-mix contained 1x Platinum®Taq Buffer (Invitrogen), 2.5 mM of MgCl_2_ (Invitrogen), 0.3 µmM of each primer, 0.1 µmM of probe, 0.2 mM Mix of dNTPs (Invitrogen), 1 U Platinum®Taq DNA polymerase (Invitrogen). Cycler conditions were as follows: predenaturation (95°C for 15 min), 40 amplification cycles (95°C for 30 s, 60°C for 30 s, 72°C for 60 s) and final extension (72°C for 10 min). Real-time amplification was run on a Stratagene MX3000 (Agilent Technology, Waldbronn, Germany). For sequencing the PCR products were run on a 1.5% agarose gel containing ethidium bromide. Images were captured on the E.A.S.Y. RH-3 (Herolab, Wiesloch, Germany) gel documentation system.

Amplicons were purified through the MSB® Spin PCRapace kit (Invitek, Berlin, Germany) and sequenced with the BigDye Terminator v3.1 Cycle Sequencing kit on an ABI Genetic Analyzer 3500 xL Dx automated sequencer (Applied Biosystems, Darmstadt, Germany). The corresponding PCR primers and reaction conditions were used in accordance with the manufacturer’s protocol.

Detection of virus nucleic acid fragments in the pooled organ tissue was followed by separate RNA extraction and cDNA synthesis of the respective bat’s particular organs. Subsequently, viral copy numbers for every organ were determined by real-time PCR and retrieved amplification products were sequenced as described above. For the phylogenetic analysis of the partial RNA-dependent RNA polymerase (RdRP) gene (L1 segment), the generic nested PCR assay previously published by Wellehan et al. was used [21]. Amplified products were visualized and sequenced as described.

Random PCR amplification of reoviral RNA from purified virus particles was performed as described by Victoria et al. [22]. Additionally, the retrieved PCR products were cloned into a TOPO TA Cloning Kit® 2.1 Vector System (Invitrogen) to reveal single sequences. The products from the cloning colony PCR were run on a 1.5% agarose gel containing ethidium bromide. Images were captured on the E.A.S.Y. RH-3 (Herolab) gel documentation system and sequenced with the corresponding colony PCR primers.

Sequence analysis and annotation were used to characterize the S1 segment and determine the serotypes of the three novel MRV strains isolated in this study [14], [17], [23].

### Virus Propagation and Purification

For random PCR, electron microscopy and whole genome sequencing, as well as propagation and purification of the isolate T3/Bat/Germany/342/08, Vero E6 cells were infected at a Multiplicity of Infection of 0.5 and incubated at cultivation conditions (5% CO_2_, 37°C). Seven days after the infection of cells, viruses were harvested and purified by low speed centrifugation (1,000×*g*; 10 min) after three freeze-and-thaw cycles. Afterwards particles were purified by ultracentrifugation (Beckman Ultrafuge, 45 h, room temperature, 100,000×*g*) through a CsCl gradient (37% CsCl in 0.05 M Tris-HCl, η_D_ = 1.3671). A white band containing virus particles became visible, which was separated and subsequently used.

### Whole Genome Sequencing

Purified RNA was transcribed and amplified utilizing the TransPlex Whole Transcriptome Amplification 2 Kit (Sigma-Aldrich). The resulting double-stranded DNA was then measured with the Qubit (Life Technol., Darmstadt, Germany) instrument, and 500 ng were used to generate a Titanium Rapid-Library (Roche, Mannheim, Germany). The library was prepared and sequenced on a 454 FLX instrument (Roche) according to the manufacturer’s instructions. Sequencing resulted in 35,036 reads and 6,032,630 bases. The sequencing data was used to perform a hybrid assembly using Newbler 2.5 (Roche) and Geneious Pro programm package 5.4 software (Biomatters Ltd., Auckland, New Zealand).

### Sequence Analysis

The nucleotide (nt) sequence contigs were analyzed with the Mega 4 (http://www.megasoftware.net) and the Geneious Pro program package 5.4 (Biomatters Ltd., Auckland, New Zealand). The identity of the nucleotide fragments was confirmed by BLASTx homology search (http://www.ncbi.nlm.nih.gov/BLAST/) against the non-redundant protein database of NCBI. For genome annotation the nt sequence was translated to amino acids (aa) in six frames with the Geneious Pro package. The sequence was also analyzed by the FGENESV Trained Pattern/Markov chain-based viral gene prediction program (http://www.softberry.com).

### Phylogenetic Tree Reconstruction

Multiple alignments (ClustalW) of nucleotide sequences were calculated with the Mega5 program (http://www.megasoftware.net). Phylogenetic tree reconstructions were based on the complete four S segments and the complete L1 segment, respectively. A complementary calculation for the partial L1 segment (197 nt, assay by Wellehan et al.) was performed that included all available, but shorter MRV sequence data from GenBank. For all calculations MrBayes v3.1.2 [24] was used, following a first model selection by using jModelTest [25], and model GTR+ G (gamma distribution) was selected for all alignments. The calculation parameters were as follows: number of runs: four, number of generations: 10,000,000 (partial L1 segment: 20,000,000), sample frequency: 100 and burn in: 25%. The results were finally visualized by the FigTree v1.2.1 program (http://tree.bio.ed.ac.uk/), a graphical viewer of phylogenetic trees.

## Results and Discussion

### Virus Isolation and Visualization

**Figure 1:**
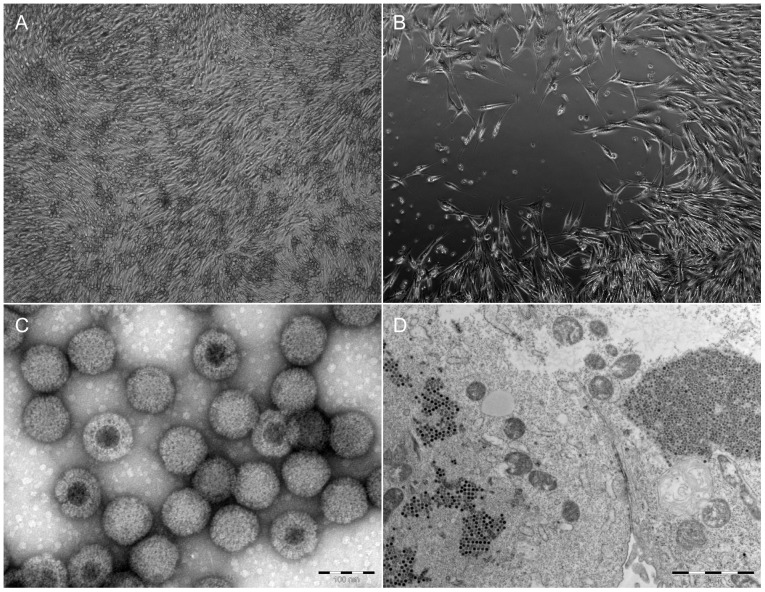
Light microscopy images of Vero E6 cells. A: control cells. B: cytopathogenic effect of infected cells 3 dpi with strain T3/Bat/Germany/342/08 (see also life cell imaging video Fig S1). Electron microscopy images of strain T3/Bat/Germany/342/08. C: Negative staining of cell-culture supernatant. Non-enveloped reoviral particles with double-layered capsid structure were observed (diameter = approximately 70 nm). Bar is indicating 100 nm. D: Ultra-thin sections of infected Vero E6 cells displayed typical contrast-rich virus particles, organized as paracrystalline structures within the cytosol. Bar is indicating 2 µm.

After inoculation and passaging of two different Vero cell lines (E6 and B4) with pooled homogenized organ tissues from 120 bats, a CPE was clearly visible on Vero E6 cells (bat 342/08) and Vero B4 cells (bats 019/09 and 021/09), respectively (Fig. 1 A and B). Cryolysates of infected cells and supernatant were purified by CsCl gradient ultracentrifugation, resulting in a distinct white band containing virus particles between η_D_ = 1,365 and η_D_ = 1,373. Virus particles were visualized by negative staining electron microscopy, revealing typical inner and outer icosahedral, non-enveloped capsids of approximately 70 nm in diameter which is characteristic for reoviruses (Fig. 1C). Ultra-thin sections of infected Vero E6 cells displayed typical electron-dense virus particles organized in a paracrystalline pattern within the cytoplasm (Fig. 1D). Life cell imaging shows clearly the developing CPE caused by T3/Bat/Germany/342/08 in Vero E6 cells compared to the negative control (Fig S1).

### Molecular Biology

**Figure 2:**
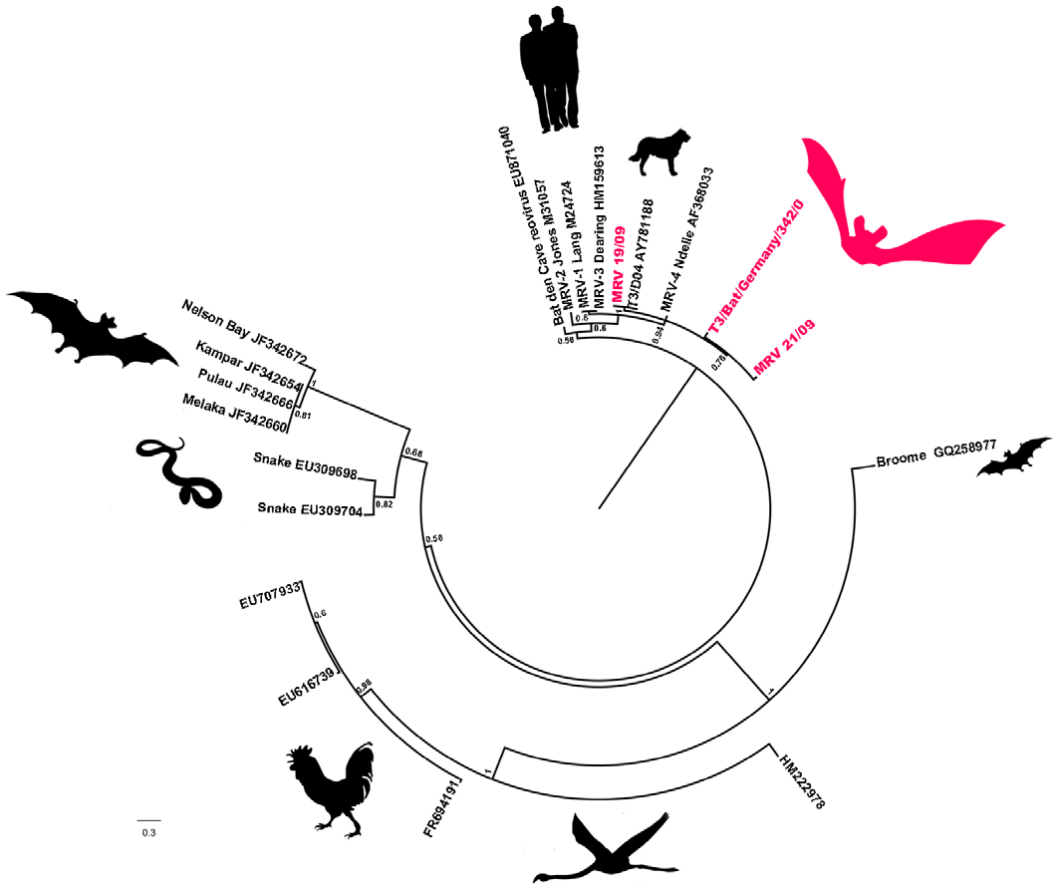
Phylogenetic tree containing the three novel isolates. Phylogenetic (Bayesian) analysis of T3/Bat/Germany/342/08, Bat MRV 019/09 and Bat MRV 021/09, using free-end alignments of nucleotide sequences of the partial L1 segments of multiple orthoreovirus species. Posterior probability values are depicted. Strains T3/Bat/Germany/342/08, Bat MRV019/09 and Bat MRV021/09 are shown in magenta and bold. Scale bar shows the evolutionary distance of 0.3 nt substitutions per position. The calculations were unrooted, but for visualization the species *reptilian orthoreovirus* was used as an outgroup.

The identification of the detected virus isolates of the family Reoviridae was verified by a generic nested PCR assay [21]. The obtained sequences of the partial RdRP gene (197 bp) indicated that these viruses represented novel strains within the species MRV of the genus *Orthoreovirus* and were tentatively named Bat MRV342/08 (JQ412755), Bat MRV019/09 (JQ412765) and Bat MRV021/09 (JQ412766) (Fig. 2). Sequence homologies between the three novel strains isolated in this study, based on the partial RdRP fragments, are given in table 1. Subsequently, isolate Bat MRV342/08 was chosen for further sequence acquisition due to the interesting pathological findings of the bat host (moderate hemorrhagic enteritis; table 2). The following random amplification, cloning and sequencing resulted in four different sequence contigs based on 20 single overlapping sequences. In parallel, extracted RNA was applied to next generation sequencing technology. Following 454 sequencing, the reads were mapped to MRV-3 Dearing as reference sequences, resulting in 10 contigs of 3,131 reads with a total of 1,054,029 bases. Only 636 bases (2.8%) had a log quality value lower than Q39. The missing terminal sequences of incomplete segments were determined by rapid amplification of cDNA ends (RACE) PCR (table 3) and revealed consensus sequences of all segments of 5′-GCUAh…yUCAUC -3′ (h = A, U or C; y = C or U). The complete genome sequences of all segments (L1–L3, M1–M3, S1–S4) were submitted to GenBank (JQ412755–JQ412764). The terminal sequences of the orthoreovirus genome segments are fairly conserved among each species and clearly differ from other orthoreovirus genera [26]. These sequences are used in positive-sense orientation as a phylogenetic marker for virus type separation [10]. All terminal sequences for strain Bat MRV342/08 proved to be similar to the MRV species within the genus *Orthoreovirus*. These conserved segment ends are typical of MRV, confirming the MRV character of Bat MRV342/08. They represent the end or start of the 5′- and 3′-UTR. The variable length at the 5′-UTR is reported to vary in all MRVs from 13 bp to 32 bp, and to range on the 3′-UTR between 35 bp and 83 bp [27]. The same range was observed for Bat MRV342/08: ranges between 12 bp in L2 to 32 bp on the S4 segment and the 3′UTR from 31 bp in the L1 segment to 80 bp in the M1 segment (table 3).

**Table 1:** Sequence homology in percent between three novel orthoreovirus strains.

Virus strain	T3/342/08	019/08	021/09
T3/Bat/Germany/342/08	100%		
Bat MRV019/09	93%	100%	
Bat MRV021/09	88%	89%	100%

Based on sequences (197 nt) revealed by nested PCR amplification, targeting the RNA-dependent RNA polymerase gene. Accession numbers: JQ412755, JQ412765 and JQ412766.

**Table 2:** Summary of the molecular biological and histopathologic results of vespertilionid bats found positive for orthoreoviruses.

Bat	Bat species/age and sex	Virus strain	Origin of bat	Positive organs	Virus load*	Pathological changes	Auto-lysis
E342/08	*P. auritus* Subadult/m	T3/Bat/Germany/342/08	Bavaria	Intestine	2.4*10^4^	Lung: non-suppurative interstitial pneumonia (++);intestine: hemorrhagic enteritis (+++)	−
E019/09	*M. mystacinus* Juvenile/f	Bat MRV 019/09	Bavaria	Intestine	1.0*10^3^	Lung: non-suppurative interstitial pneumonia (+)	−
E021/09	*M. mystacinus* Juvenile/f	Bat MRV 021/09	Bavaria	Intestine	1.3*10^4^	Lung: non-suppurative interstitial pneumonia (+)	−
E022/09	*M. mystacinus* Juvenile/f	Bat MRV 021/09	Bavaria	Intestine Lung	1.0*10^3^ 8.7*10^4^	Lung: non-suppurative interstitial pneumonia (+++);spleen: follicular hyperplasia (+); intestine:coccidiosis (+++)	+
E099/09	*P. pipistrellus* Adult/m	Bat MRV 021/09	Bavaria	Liver Heart Brain	1.3*10^3^ 3.5*10^3^ 2.4*10^4^	Lung: alveolar edema (++)	−
E121/09	*P. nathusii* Subadult/m	Bat MRV 021/09	Bavaria	Intestine Liver	1.0*10^3^ 2.4*10^4^	Lung: alveolar edema (+++), non-suppurative interstitial pneumonia (+), leucocytostasis of blood vessels(+++);spleen: follicular hyperplasia (+++); kidney: glomerulopathy(+++)	−
E126/09	*P. kuhlii* Adult/m	Bat MRV 021/09	Bavaria	Intestine	1.9*10^3^	Lung: non-suppurative interstitial pneumonia (++)	+
E131/09	*N. noctula* Adult/f	Bat MRV 021/09	Bavaria	Intestine	1.0*10^3^	Spleen: follicular hyperplasia (++); intestine: catarrhal-hemorrhagic enteritis (++); enteritis (+)	++

m, male; f, female; −, none; +, mild; ++, moderate; +++, severe (degree of severity);

* per ml of homogenized organ tissue (average size of tissue piece 8 mm^3^).

**Table 3:** Terminal sequences and size of the ten segments of the T3/Bat/Germany/342/08 genome.

		5′ end		ORF/Protein				3′ end	
Segment	Segment size [nt]	Terminal sequence	UTR [nt]	region [nt]	size [aa]	class	Predicted function+	UTR [nt]	Terminal sequence
L1	3,853 bp	**GCUA**CA	18 bp	19…3,822	1,267	λ3	RNA-dependent RNApolymerase	31 bp	AC**UCAUC**
L2	3,915 bp	**GCUA**UU	12 bp	13…3,882	1,289	λ2	Guanyltransferase, methyltransferase	74 bp	*AU**UCAUC**
L3	3,901 bp	**GCUA**AU	13 bp	14…3,841	1,275	λ1	RNA binding, NTPase, helicase, RNA triphosphatase	60 bp	AC**UCAUC**
M1	2,303 bp	**GCUA**UU	13 bp	14…2,224	736	µ2	Binds RNA NTPase	80 bp	CU**UCAUC**
M2	2,197 bp	**GCUA**AU*	30 bp	31…2,157	708	µ1	Cell penetration, transcriptase activation	40 bp	AA**UCAUC**
M3*	2,241 bp	**GCUA**AA*	18 bp	19…2,184	721	µNS	Unknown NS	57 bp	AU**UCAUC**
S1	1,416 bp	**GCUA**UU	12 bp	13…1,380 71…433	455 120	σ1 σ1s	Cell attachment Unknown NS	36 bp	UU**UCAUC**
S2	1,331 bp	**GCUA**UU	18 bp	19…1,275	418	σ2	Binds dsRNA	56 bp	AU**UCAUC**
S3	1,198 bp	**GCUA**AA*	27 bp	28…1,128	366	σNS	Inclusion formation, binds ssRNA	70 bp	AA**UCAUC**
S4	1,196 bp	**GCUA**UU	32 bp	33…1,130 785…567§ 1,030…800§	365 73 68	σ3 σ3a σ3b	Binds dsRNA Unknown function Unknown function	66 bp	*AU**UCAUC**

Functions of proteins encoded by open reading frames in between the non-coding sequences are predicted analog to known members of the genus Orthoreovirus. bp, base pair;

* Terminal sequence determined by RACE PCR;

+ Predicted protein functions reported by Coombs, 2006;

§ reversely directed ORF with unknown function.

Agarose gel electrophoresis was used to examine the migration patterns of the ten segments of Bat MRV342/08. The S1 segment of Bat MRV342/08 was found to migrate with delay, a typical property of serotype 3 MRV strains [28].

### Phylogeny and Characterization

After revealing the full genome sequence of one of the novel orthoreovirus strains (Bat MRV342/08), a more in-depth molecular characterization was performed, highlighting some special features in comparison to other strains.

**Figure 3:**
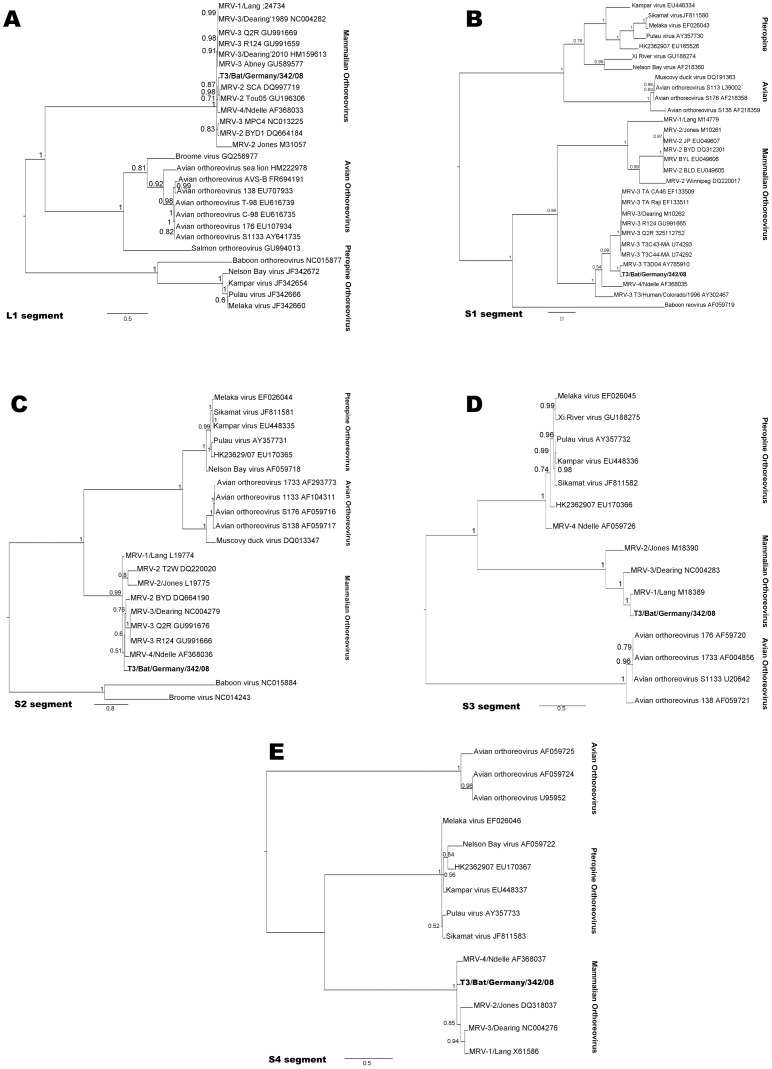
Phylogenetic trees of five particular segments of isolate Bat/342/08. Bayesian analysis of T3/Bat/Germany/342/08 using free-end alignments of nucleotide sequences of five segments of multiple orthoreovirus species. In genome order: A: complete L1 segment. B: S1 segment, complete cds, C: complete S2 segment, D: complete S3 segment, E: complete S4 segment, Posterior probability values are depicted. Strain T3/Bat/Germany/342/08 is shown in bold. Scale bar shows the evolutionary distance of nt substitutions per position. The calculations were unrooted, but for visualization mid-point root was applied.

Phylogenetic tree reconstructions by Bayesian and maximum likelihood analyses were based on the nucleotide sequence of the complete L1, S2, S3, and S4 segments (Fig. 3A; Fig. 3C–E). Commonly, orthoreoviruses are taxonomically classified on the basis of the S1 segment [14], [23]. We based the phylogenetic reconstruction of the S1 segment on the complete ORF (nt) encoding for the σ1 protein (Fig. 3B). The topology of this phylogenetic tree assigned strain Bat MRV342/08 to serogroup 3 within the species MRV and the strain was renamed as T3/Bat/Germany/342/08. Strain T3/Bat/Germany/342/08 appears to be monophyletic and forms a distinct lineage together with the canine strain T3D/04 (Fig. 3B). In every calculation the segments S2, S3 and S4 of T3/Bat/Germany/342/08 were shown to cluster within the species MRV, without a distinct relation to a single serotype or –group (Fig. 3C–E). However, based on the L1 segment, the reconstructed tree assorted strain T3/Bat/Germany/342/08 to serogroup T2/Jones within the species MRV (Fig. 3A). Alignments and calculations for the remaining reo-viral segments (M1–3, L2–3) are based on an insufficient number of sequences, due to the unavailability of comparative sequence data from GenBank (data not shown).

Pairwise nucleotide and deduced amino acid comparison between strain T3/Bat/Germany/342/08 and the prototype strains T1 (MRV-1 Lang), T2 (MRV-2 Jones), T3 (MRV-3 Dearing) and T4 (MRV-4 Ndelle) was performed for all ten segments (table 4). The predicted protein-coding ORFs were mostly similar in length and had a similar start and stop position compared to the previously reported ORFs of the prototype strains T1/Lang, T2/Jones, T3/Dearing and T4/Ndelle (table 4).

**Table 4:** Comparison of nucleotide and amino acid sequences of the prototype strains available for strain T3/Bat/Germany/324/08.

342/08 genes	nt (%)				342/08 proteins	aa (%)			
	T1L	T2J	T3D	T4N		T1L	T2J	T3D	T4N
L1	89.4	75.4	89.2	90	λ3	98	91.8	97.7	97.9
L2	75.2	72.7	87.3	na	λ2	92.2	86.4	97.6	na
L3	87.5	77.1	87.5	na	λ1	98.7	96	98.7	na
M1	87.7	72.2	87.4	na	µ2	96.7	81.3	95.8	na
M2	84.4	77.2	90.7	89.4	µ1	98	97.2	97.6	98.2
M3	84	84	85	na	µNS	96	83	96	na
S1	37.5	48.2	79.6	62.9	σ1	24.3	41.4	88.2	66.8
S2	86.2	76.6	84	85	σ2	98.8	94	99	97.1
S3	93.8	73.4	85.1	na	σNS	97.6	85.8	97.3	na
S4	86.9	77.4	87.7	83.4	σ3	96.7	92.6	96.2	93.6

na = not available.

The segments L1 and L3 of strain T3/Bat/Germany/342/08 showed high similarity to all MRV prototypes, with T2J being the most distant (supported by Fig. 3A). The L2 segment of strain T3/Bat/Germany/342/08 had the highest consistence with prototype T3/Dearing on both the nucleotide and deduced amino acid level. The length of this segment was the same as the one reported for strains T1 and T3, while the protein described for strain T2 was found to be reduced by one amino acid. Comparing the M segments with prototype strains resulted in different parities on nucleotide and amino acid levels. Segments M1 and M3 showed the highest identity with strains T1/Lang and T3/Dearing on the amino acid level, whereas the segment M2 appeared to be uniform with all four prototypes. On the nucleotide level M3 was similar to M2 and was uniform to all three prototype sequences available for this segment. The length of the M1 segment was identical for all strains compared. For the M2 segment only strain T4/Ndelle was found to encode for a protein longer by one amino acid. In the M3 segment the size of the protein predicted was identical to that of the other prototype strains.

The largest segments (L-segments) are encoding monocistronicly for the proteins λ1, λ2 and λ3 and the medium sized segments (M-segments) encoding the µ1, µ2 and µ3 proteins, respectively [12], [29]. The λ and µ class proteins are conserved among the orthoreovirus species. Within these segments a lesser degree of diversity was observed. The M1 and M3 segments appeared to be closely related to the prototype strains T1/Lang and T3/Dearing, while M2 showed the same relationship to all prototypes.

The highest diversity among all segments was observed for S1, which revealed the highest nucleotide and size similarity to the prototype strain T3/Dearing. Nine out of ten segments of orthoreoviruses are monocistronic; a single ORF can be identified on each segment. The S1 segment is bi- or tricistronic and overlapping ORFs are expected [30]. Among all sequences compared, the highest identity was observed for the S2 segment which agreed to 99% with T3/Dearing at the deduced amino acid level. Detailed numbers of nucleotides and deduced amino acid comparisons of all proteins are given in table 4. The four small orthoreovirus segments (S1, S2, S3 and S4) encode for the σ class proteins which play a crucial role in cell attachment, dsRNA binding, hemagglutination ability and antigenicity and are associated with the assortment of the different genome segments during virus assembly [31]. Genome analysis and phylogenetic tree reconstructions have proved that strain T3/Bat/Germany/328/08 was clearly related to the species MRV type T3. The gene constellation observed in the S1 segment (σ1 and σ1s genes) was characteristic of mammalian orthoreoviruses [23]. Together with the high homology on the nucleotide (79.6%) and the amino acid (88.2%) level and the identical length of the S1 segment compared to that of strain T3, this justified the assignment of strain T3/Bat/Germany/342/08 to serogroup 3/Dearing within the species MRV. The S1 is the most divergent segment within the orthoreovirus genome, and the encoding genes are used to assign novel viruses to different species and strains [14], [23], [32,]. The S1 segments of all known bat- and human-borne reoviruses of the species *Pteropine orthoreovirus* are tricistronic [12]. In contrast, MRVs are reported to have a bicistronic genome organization [23]. The S1 segment of strain T3/Bat/Germany/342/08 appeared to be bicistronic with one larger ORF encoding for the σ1 protein and a second smaller one within encoding for the σ1s protein. Orthoreoviruses can be divided into a fusogenic and a non-fusogenic cluster, depending on their ability to cause syncytial cell fusion in mammalian cell cultures [26]. The underlying gene function for building syncytial fusion is the FAST (fusion-associated transmembrane) protein located on the S1 segment of all orthoreoviruses (S4 segment in Broome). T3/Bat/Germany/342/08 and all other members of the species MRV do not code for the FAST protein and therefore were not fusogenic.

Segments S2 and S3, encoding for the σ2 (σA in fusogenic MRV species) and σNS proteins, were found to be similar in size and length to the corresponding proteins of MRV prototype strains T1/Lang, T2/Jones and T3/Dearing. The highest identity of all segments (99% aa) was found by sequence comparison of the S2 segment to strain T3/Dearing. The σ3 protein, located on the S4 segment, plays a crucial role in dsRNA binding, assortment and replication [31]. Interestingly, we detected three ORFs including two novel, reversely directed ORFs in the S4 segment of strain T3/Bat/Germany/342/08, although in other MRV strains this segment is monocistronic (table 3). So far, only the negative strand of the reovirus dsRNA genome has been described for transcription. The possibility of transcription and perhaps the function of two hypothetical proteins on the positive strand have to be examined in further studies.

**Figure 4:**
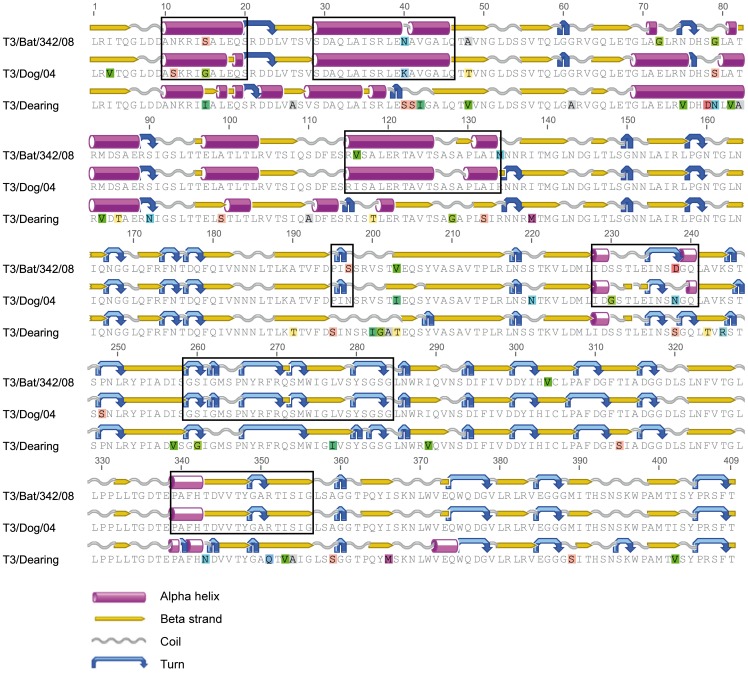
Free-end protein alignment of the partial σ1 protein. Its abridged length is due to the availability of only a shorter T3D/04 sequence. The protein structure (α-helix, β-strand, coil and turn) was predicted by Geneious Pro for all strains, and rectangles indicate structure similarity between strains T3/Bat/Germany/342/08 and T3D/04 compared to prototype strain T3/Dearing.

Due to the close phylogenetic relationship between strain T3/Bat/Germany/342/08 and the dog isolate T3D/04, both amino acid sequences were aligned in comparison to prototype strain T3/Dearing (Fig. 4). In this alignment the first 46 amino acids were missing because of the shortened T3D/04 sequence available at GenBank. A total of 46 alterations were observed. On six identical positions unique sequence differences were observed in all strains (aa positions 15, 40, 48, 79, 203 and 238). Four differences were found to be unique for strain T3D/04 (aa positions 3, 220, 230 and 248) and also four differences for strain T3/Bat/Germany/342/08 (aa positions 72, 116, 197 and 304). Remarkably, the majority of alterations (n = 32) were shared in T3/Bat/Germany/342/08 and T3D/04 but not inT3/Dearing. The protein structure (α-helix, β-strand, coil and turn) was predicted for all strains, and rectangles (Fig. 4) indicate higher structure similarities between strains T3/Bat/Germany/342/08 and T3D/04 compared to T3/Dearing.

Although the genome of strain T3/Bat/Germany/342/08 showed many characteristics of MRV-3, this was not consistent in all of the segments. Unique characteristics of strain T3/Bat/Germany/342/08 were a striking relationship to strain T1/Lang for the L1, M1 and S4 segments and the detection of two additional ORFs in the S4 segment.

### Infected Bats and Organ Tropism

To determine the viral load in individual organs of infected bats, a real-time PCR assay targeting the RNA-dependent RNA polymerase gene was developed. Reoviral RNA was confirmed in internal organs of the three bats from which the concurrent strains were isolated (summarized in table 5). Subsequently, all 120 bats investigated in this study were screened via real-time PCR for the presence of reoviral RNA. Five additional bats were found to carry strain Bat MRV021/09 with a distinct organ tropism (intestine). Detailed results including the viral load are given in table 2. No distinct correlation between infection status and age or gender of the infected bats was observed. All bats positive for reoviral RNA originated from Bavaria (8/85). The number of samples from other locations in Germany (n = 35) was not sufficient to statistically correlate reovirus infections to the bat origin. Interestingly, reovirus infections were observed in six different bat species (table 2) belonging to four different genera of vespertilionid bats: *Myotis* [n = 3], *Pipistrellus* [n = 3], *Nyctalus* [n = 1] and *Plecotus* [n = 1]. Whereas strains T3/Bat/Germany/342/08 and Bat MRV 019/09 were only identified in its initial host, strain Bat MRV21/09 was detected in five different bat species. These findings indicate a broad host range and an opportunistic character of bat orthoreoviruses, as well as a lacking of host adaptation in European bats [33]–[34].

**Table 5:** Overview: Orthoreovirus strains isolated from or associated with bats.

Strain	Year	Host	Origin	Datatype	Sequencesavailable	Cluster	Reference	#
Nelson Bay	1970	Pteropus (*Megachiroptera*)	Australia	Isolate	Genome	Pteropine ORV	Gard et al. (1973)	[4]
Pulau	1999	Pteropus (*Megachiroptera*)	Malaysia	Isolate	S1,S2,S3,S4	Pteropine ORV	Pritchard et al. (2006)	[5]
Broome	2002	Pteropus (*Megachiroptera*)	Australia	Isolate	Genome	Own cluster	Thalman et al. (2010)	[8]
Melaka	2006	Human (bat associated)	Malaysia	Isolate	S1,S2,S3,S4	Pteropine ORV	Chua et al. (2007)	[6]
Kampar	2006	Human (bat associated)	Malaysia	Isolate	S1,S2,S3,S4	Pteropine ORV	Chua et al. (2008)	[10]
HK23629/07	2007	Human (bat associated)	China	Isolate	S1,S2,S3,S4	Pteropine ORV	Cheng et al. (2009)	[9]
Xi River	2010	Rousettus (*Megachiroptera*)	China	Isolate	S1,S3	Pteropine ORV	Du et al. (2010)	[7]
Sikamat	2010	Human (bat associated)	Malaysia	Isolate	S1,S2,S3,S4	Pteropine ORV	Chua et al. (2011)	[11]
Bat den Cave	2010	Bat spp.	USA	Sequence	Partial L1	Mammalian ORV	Acc.No. EU871040	
T3/Bat/Germany/342/08	2010	Vespertilionid (*Microchiroptera*)	Germany	Isolate	Genome	Mammalian ORV	This study	
Bat MRV019/09	2011	Vespertilionid (*Microchiroptera*)	Germany	Isolate	Partial L1	Mammalian ORV	This study	
Bat MRV021/09	2011	Vespertilionid (*Microchiroptera*)	Germany	Isolate	Partial L1	Mammalian ORV	This study	

### Pathological Findings

During necropsy, five out of eight bats positive for reoviruses had mild to severe traumatic injuries. Two bats revealed moderate to severe hemorrhagic or catarrhal-hemorrhagic enteritis, respectively (table 2). Five carcasses had early signs of decomposition. Based on histological examination, single- or multi-organ inflammatory lesions were observed in seven of the eight bats. The lung was predominantly affected. Three bats revealed additional activation of the lymphoreticular tissue of the spleen. Although two of eight bats revealed signs of hemorrhagic enteritis and six bats had mild to severe interstitial pneumonia the impact of reovirus infection on bat hosts remains unclear.

### Relationship to T3D/04

Strain T3/Bat/Germany/342/08 shared a monophyletic relationship (95% nt/96% aa) with strain T3D/04 isolated from a dog with a parvovirus type 2 co-infection, showing hemorrhagic enteritis [15]. Interestingly, the *P. auritus* bat infected with strain T3/Bat/Germany/342/08 (n = 1/1) and the *N. noctula* bat infected with Bat MRV021/09 (n = 1/5) also showed hemorrhagic enteritis (table 2). Attention should be drawn to the recent description of an adenovirus isolated from European bats and its closest viral relative which caused severe disease in canids [18]. The recent report discussed the evolutionary bat-origin of the canine adenoviruses and an inter-species transmission event between bats and dogs. Compared to these adenoviruses the relationship between the bat-reovirus (T3/Bat/Germany/342/08) and the dog-reovirus (T3D/04) is even more distinct (with 95% nt/96% aa) on the S1 segment and on the partial L1 segment. Moreover, they form a clearly separate lineage within the T3 MRVs. The alignment and σ1-protein structure prediction (Fig. 4) of strains T3/Bat/Germany/342/08 and T3D/04 displayed similar protein structure patterns, in contrast to the prototype strain T3/Dearing. Uniquely, both strains shared the majority of alterations on the σ1 protein (viral haemagglutinin) which has been demonstrated in mice experiments to play a crucial role in reovirus pathogenesis [30]. The structure identity observed in the σ1 protein among reoviral strains T3/Bat/Germany/342/08 and T3D/04 suggests an evolutionary relatedness. An extensive phylogenetic analysis of all segments of both reoviral strains might provide further evidence for a past inter-species transmission or a shared but yet unknown common ancestor reovirus. Unfortunately, no further sequence data for strain T3D/04 are available at GenBank.

In conclusion, we demonstrate the presence of three novel orthoreoviruses in eight bats of six different vespertilionid species from Europe, suggesting a broad distribution of orthoreoviruses in chiropteran species. In contrast to other bat-borne reoviruses assigned to PRV the European strains belong to MRVs with the highest similarity to MRV-3. MRVs are able to infect nearly every class of mammals including man [12]. The distinct tropism of the novel viruses to the bats’ intestines indicates a fecal-oral transmission route. Although MRVs are known to cause rather mild and unapparent infections, recent studies also reported severe disease outcomes in a dog and man [15]–[17], [35]. Considering that reovirus-infected bats were found in close proximity to humans, a possible spill-over and zoonotic potential of bat-borne MRV needs to be discussed.

## Supporting Information

Figure S1 **Life-cell video of Vero E6 cells infected with strain Bat/342/08.** Left, Vero E6 cells control. Right, Vero E6 cells infected with T3/Bat/Germany/342/08 (MOI 0.1). Images were captured every 15 minutes for a period of 72 hours.
(AVI)
